# rMAP 2.0: a modular, reproducible, and scalable WDL–Cromwell–Docker workflow for genomic analysis of ESKAPEE pathogens

**DOI:** 10.1093/bioadv/vbag046

**Published:** 2026-02-13

**Authors:** Gerald Mboowa, Ivan Sserwadda, Stephen Kanyerezi

**Affiliations:** The African Centre of Excellence in Bioinformatics and Data-Intensive Sciences, P.O Box 7062, Kampala, Uganda; The Infectious Diseases Institute, College of Health Sciences, Makerere University, P.O Box 22418, Kampala, Uganda; The African Centre of Excellence in Bioinformatics and Data-Intensive Sciences, P.O Box 7062, Kampala, Uganda; The Infectious Diseases Institute, College of Health Sciences, Makerere University, P.O Box 22418, Kampala, Uganda; The African Centre of Excellence in Bioinformatics and Data-Intensive Sciences, P.O Box 7062, Kampala, Uganda; The Infectious Diseases Institute, College of Health Sciences, Makerere University, P.O Box 22418, Kampala, Uganda

## Abstract

**Motivation:**

Antimicrobial resistance surveillance in ESKAPEE pathogens (*Enterococcus faecium*, *Staphylococcus aureus*, *Klebsiella pneumoniae*, *Acinetobacter baumannii*, *Pseudomonas aeruginosa*, *Enterobacter* spp., and *Escherichia coli*) requires reproducible, portable whole-genome analysis that public health laboratories including those operating under data-sovereignty constraints can run on laptops, institutional servers, or cloud backends without local dependency conflicts. rMAP 2.0 addresses these needs using a containerized Workflow Description Language pipeline executed with Cromwell.

**Results:**

rMAP 2.0 standardizes end-to-end bacterial whole-genome analysis—read quality control, trimming, assembly and annotation, resistance/virulence/mobile-element profiling, sequence typing, pangenome inference, and phylogenetic reconstruction using containerized execution, and generates a single interactive HTML report that collates outputs for rapid review. The workflow supports fully offline execution (including BLAST searches) for data-sovereign deployments and can run on local workstations, institutional servers, and cloud backends where Docker is supported, providing a consistent execution environment without local tool installation. In a representative benchmark of 20 Enterobacterales isolates, rMAP 2.0 completed a cohort run in ∼4.5 hours on an 8-core/16-GB laptop and flagged a public record misannotated in public repository metadata (SRR9703249, reclassified from *K. pneumoniae* to *Enterobacter cloacae* sequence type 182), while confirming lineage assignments such as *E. coli* sequence type 131.

**Availability and implementation:**

https://github.com/gmboowa/rMAP-2.0 and example workflow reports are available at: https://gmboowa.github.io/rMAP-2.0/

## 1 Introduction

The rise of antimicrobial resistance (AMR) among healthcare-associated bacterial pathogens is undermining the effectiveness of antibiotics and increasing morbidity, mortality, and costs worldwide. A widely used practical grouping of these high-burden organisms is the ESKAPEE pathogens which includes *Enterococcus faecium*, *Staphylococcus aureus*, *Klebsiella pneumoniae*, *Acinetobacter baumannii*, *Pseudomonas aeruginosa*, *Enterobacter* spp., and *Escherichia coli*, which collectively account for a substantial fraction of hospital-acquired infections and frequently exhibit multidrug resistance (De Oliveira *et al.* 2020, Ntim *et al.* 2025). Importantly, ESKAPEE is an operational grouping rather than a World Health Organization (WHO) taxonomy; however, there is strong overlap with global AMR frameworks. Several members of this group are monitored in WHO’s Global Antimicrobial Resistance and Use Surveillance System (GLASS), and many are designated as Critical or High priority organisms in the WHO Bacterial Priority Pathogens List (2024), underscoring their public health importance and the need for scalable, standardized genomic analysis for surveillance and response. Healthcare-associated infections (HAIs) caused by ESKAPEE bacteria account for nearly 70% of HAIs (de Paula *et al.* 2025). Among these drug-resistant pathogens, *E. coli*, *K. pneumoniae*, *P. aeruginosa*, and *A. baumannii* are listed in WHO GLASS (2022), while *E. faecium*, *S. aureus*, *K. pneumoniae*, *A. baumannii*, *P. aeruginosa*, and *Enterobacter* species are classified as critical or high-priority organisms in the 2024 WHO Bacterial Priority Pathogens List (2024).

Whole genome sequencing (WGS) has emerged as an indispensable tool for pathogen characterization and monitoring AMR (Köser *et al.* 2014). During the COVID-19 pandemic, many national public health laboratories in low- and middle-income countries (LMICs) acquired next-generation sequencing (NGS) platforms and developed foundational genomic capacity (Köser *et al.* 2014, Pronyk *et al.* 2023, Getchell *et al.* 2024, Mboowa *et al.* 2024, Márquez *et al.* 2025, Praharaj et al. 2025). However, implementing comprehensive bioinformatics pipelines in these settings remains a significant challenge due to limited computational infrastructure, complex software dependencies, and difficulties maintaining and updating workflows on local computational infrastructure (Carlos *et al.* 2020).

The rapid expansion of sequencing data, combined with the growing complexity of microbial genomic analysis, has driven a paradigm shift toward adopting cloud-native bioinformatics resources (Stein 2010, Dai *et al.* 2012, Grossman 2019, Hanna and Latha 2024). The rapid expansion of genomic surveillance generates continuously growing datasets, often ranging from terabytes to petabytes, a scale that cloud platforms are designed to handle through elastic computing and on-demand, durable storage. Their integration with major public repositories (e.g. SRA/ENA) reduces data-transfer bottlenecks and shortens turnaround times. Containerized pipelines and Workflow Description Language (WDL) executed by engines such as Cromwell strengthen reproducibility, provenance tracking, and cross-institutional portability. In addition, robust security controls and audit capabilities can support compliance with clinical data-protection frameworks such as the Health Insurance Portability and Accountability Act (HIPAA) and the General Data Protection Regulation (GDPR) when appropriately configured, making cloud computing a secure and cost-effective complement to traditional high-performance computing platforms.

Implemented within a WDL–Cromwell–Docker framework, rMAP 2.0 harnesses this ecosystem to ensure portable, reproducible, and scalable whole-genome analyses of ESKAPEE pathogens. This architecture not only standardizes workflow execution across local, HPC, and cloud environments but also simplifies deployment in resource-limited settings by encapsulating dependencies within containers. By reducing technical barriers and promoting interoperability, rMAP 2.0 supports near–real-time, population-scale genomic surveillance, thereby accelerating the translation of sequencing data into actionable insights for public health decision-making.

Our initial release, rMAP 1.0 (Sserwadda and Mboowa 2021), required users to manually install and manage a broad suite of bioinformatics tools and dependencies within a Conda environment. However, as tool versions evolved and dependencies diverged, maintaining compatibility became increasingly challenging, often compromising the reliability of pipeline execution.

Web-based platforms such as Microreact (https://microreact.org/) offer powerful capabilities for analysis and interactive visualization of pathogen genomic data. However, their dependence on online data submission introduces significant privacy and confidentiality risks, limiting adoption in public health laboratories and clinical settings where sensitive genomic and epidemiological data may not be transferred to external servers. In contrast, rMAP 2.0 delivers secure, end-to-end genomic analysis and visualization within local or institutional computing environments, providing a scalable and reproducible solution that fully aligns with stringent data governance requirements.

**Figure 1:**
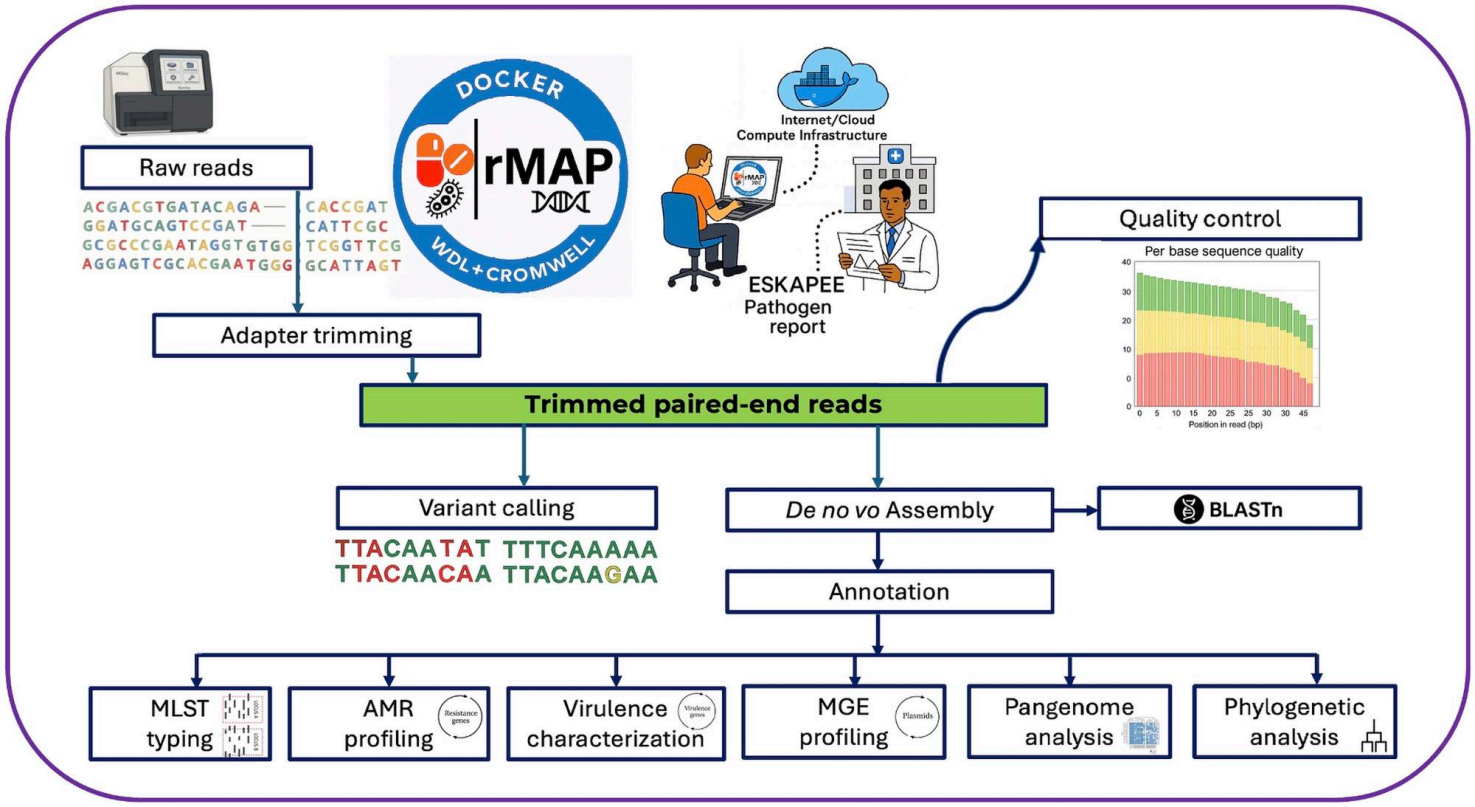
Workflow description of rMAP-2.0. Raw paired-end reads (e.g. from an Illumina MiSeq) undergo adapter trimming, yielding trimmed reads, that go through quality control. The trimmed reads feed two analysis paths: (i) reference-based variant calling and (ii) *de novo* assembly, followed by annotation and BLAST. Downstream modules perform MLST typing, antimicrobial-resistance (AMR) gene profiling, virulence-factor characterization, mobile genetic element (MGE) profiling (including plasmids), pangenome analysis, phylogenetic inference, and tree visualization. Outputs from all modules are collated into a single consolidated (merged) interactive HTML report for rapid review and sharing. Abbreviations: AMR, antimicrobial resistance; ESKAPEE, *Enterococcus faecium, Staphylococcus aureus, Klebsiella pneumoniae, Acinetobacter baumannii, Pseudomonas aeruginosa, Enterobacter* spp., *Escherichia coli*; MGE, mobile genetic elements; MLST, multilocus sequence typing; WDL, workflow description language.

To address critical challenges of privacy, reproducibility, and scalability, we developed rMAP 2.0, a modular microbial genomics pipeline written in the WDL and executed by Cromwell with Docker containerization (Fig. 1). Each module runs in a version-pinned container, removing the need for local installations and ensuring consistent performance across workstations, HPC systems, and cloud platforms. On first use, rMAP 2.0 automatically retrieves and caches the necessary containers; subsequent runs can proceed offline. Remote BLAST searches against NCBI are supported when internet access is available, while fully offline analyses can be performed using local BLAST databases for ESKAPEE pathogens. Its cloud-native architecture supports agile updates as tools and reference datasets evolve, modules are independently updatable, provenance is tracked, and releases are packaged for automated deployment, strengthening reproducibility and long-term sustainability. rMAP 2.0 is portable across local, HPC, and cloud infrastructures (e.g. Terra), leverages Dockstore-hosted containers, and supports fully on-premises execution, ensuring that sensitive genomic and epidemiological data remain within institutional boundaries. This design delivers a timely and practical solution for secure, population-scale microbial genomic surveillance.

## 2 Methods

### 2.1 Workflow implementation and deployment

To support flexible deployment and safeguard user’s data sovereignty, rMAP 2.0 is implemented as a WDL/Cromwell workflow that executes in fully containerized environments. The same pipeline runs seamlessly on cloud platforms, institutional HPC clusters, or personal workstations using versioned container images referenced on Dockstore (https://hub.docker.com/). Because inputs and outputs can be mounted locally, all analyses can be performed entirely on-premises, eliminating the need to transfer genomic data to third-party computer servers. rMAP 2.0 accepts raw Illumina paired-end FASTQ reads and GenBank or Fasta reference genomes as inputs (Table 1), with all analytic modules executed within versioned Docker images (Table 2) that are automatically retrieved and cached from Dockstore on first use.

**Table 1:** Key entry-point parameters.

Input name	Type	Required	What it controls/notes	Example value
**input_reads**	Array [File]	Yes	Paired-end FASTQ files (R1/R2 per sample, interleaved in the array).	[“/path/SRR8753739_1.fastq.gz”, “/path/SRR8753739_2.fastq.gz”, “…”]
**adapters**	File	Yes (if trimming)	Adapter FASTA used by Trimmomatic (ILLUMINACLIP).	/path/adapters.fa
**reference_genome**	File	Yes (variant calling)	Reference genome used for read mapping and variant calling.	/path/reference.gbk
**reference_type**	String	Yes (if reference provided)	Reference format: “genbank” or “fasta.”	genbank
**do_trimming**	Boolean	No	Enable/disable Trimmomatic trimming.	True
**quality_encoding**	String	No	FASTQ quality encoding for Trimmomatic (phred33 or phred64).	phred33
**do_quality_control**	Boolean	No	Enable/disable FastQC and MultiQC summaries.	True
**do_assembly**	Boolean	No	Enable/disable de novo assembly (MEGAHIT).	True
**do_variant_calling**	Boolean	No	Enable/disable variant calling (Snippy). Requires reference_genome.	True
**do_annotation**	Boolean	No	Enable/disable annotation (Prokka).	True
**do_amr_profiling**	Boolean	No	Enable/disable AMR profiling (ABRicate).	True
**do_reporting**	Boolean	No	Generate consolidated interactive HTML report.	True
**max_cpus**	Int	No	Maximum vCPUs per task.	8
**max_memory_gb**	Int	No	Maximum RAM (GB) per task.	16

This table summarizes the primary inputs most users need to run rMAP-2.0. The Required column indicates whether a parameter must be provided for successful workflow submission under the relevant run mode: Yes denotes a mandatory input, Yes (if …) denotes an input required only when the corresponding module/option is enabled, and No denotes an optional input that can be omitted or left at its default. For example, input_reads is required (Yes) because paired-end Illumina FASTQ files are necessary for execution; when starting from SRA accessions, reads can be retrieved and converted to FASTQ using standard tools (e.g. SRA Toolkit prefetch/fasterq-dump or equivalent) and then supplied via the input JSON. Extended option lists and module-specific parameters (including BLAST options, local database paths, and pangenome/phylogeny settings) are provided in the GitHub repository documentation (https://github.com/gmboowa/rMAP-2.0).

**Table 2:** Tools used in rMAP-2.0 with Docker images.

Step	Tool	Docker image
**Trimming**	Trimmomatic	staphb/trimmomatic: 0.39
**Quality control**	FastQC	staphb/fastqc: 0.11.9
**Assembly**	MEGAHIT	quay.io/biocontainers/megahit: 1.2.9—h5ca1c30_6
**Annotation**	Prokka	staphb/prokka: 1.14.6
**Variant calling**	Snippy	staphb/snippy: 4.6.0
**Sequence typing**	MLST	staphb/mlst: 2.19.0
**Pangenome analysis**	Roary	gmboowa/roary-pillow: 0.4
**Phylogenetic inference**	FastTree	staphb/fasttree: 2.1.11
**Tree visualization**	ETE3	gmboowa/ete3-render: 1.18
**AMR profiling**	ABRicate	staphb/abricate: 1.0.0
**MGE profiling**	ABRicate	staphb/abricate: latest
**Virulence profiling**	ABRicate	staphb/abricate: latest
**Similarity search**	BLAST+	gmboowa/blast-analysis: 1.9.4

### 2.2 Workflow comparison and benchmarking

We contextualized rMAP-2.0 against Bactopia (Petit and Read 2020), a widely used modular bacterial WGS workflow, alongside representative web platforms and visualization tools (Table 3). This highlights a key practical distinction: rMAP-2.0 and Bactopia are offline-capable, containerized workflows that can run on local/HPC/cloud resources without requiring users to upload data, whereas several alternative platforms operate primarily as hosted services that require data upload and/or provide only partial analysis components (e.g. visualization without inference).

**Table 3:** Feature comparison of rMAP 2.0 vs selected platforms.

Platform	Offline/online	Containerization	Need to upload data?	AMR	MLST/cgMLST	Phylogeny	Pangenome	Visualization/reporting	Target users
**rMAP 2.0**	Offline-capable (local/HPC/cloud via WDL/Cromwell); reports can be hosted online	Yes (Docker + WDL/Cromwell)	No (runs on local/HPC/cloud inputs)	Yes	Yes	Yes	Yes	Interactive HTML reports (QC, assembly, MLST, AMR, etc.)	Public health labs and researchers needing reproducible workflows
**Pathogenwatch**	Online web platform	No (hosted service)	Yes	Yes	Yes (species-dependent schemes)	Yes	Limited/no	Interactive web reports and visualizations	Public health surveillance users
**BV-BRC/PATRIC**	Online web platform (private workspace)	No (hosted service)	Yes	Yes	Yes (incl. cgMLST services)	Yes	Partial/limited	Web viewers (trees, genome browser, etc.)	Researchers needing integrated databases
**EnteroBase**	Online web platform	No (hosted service)	Yes	Yes (species-dependent)	Yes (MLST, cgMLST, wgMLST; clustering)	Yes	Limited/no	Web exploration of population structure and metadata	Epidemiologists and population genomics researchers
**Microreact**	Online visualization platform; can be self-hosted	Not a bioinformatics pipeline	Yes (upload tree + metadata)	No	No	Displays user-provided trees	No	Interactive visualization (maps/trees/timelines)	Teams sharing genomic epidemiology results
**Bactopia**	Offline-capable (local/HPC/cloud via Nextflow)	Yes (Docker/Singularity)	No	Yes	Yes	Yes	Yes	Pipeline outputs and summaries (not primarily a web dashboard)	Bioinformatics teams needing comprehensive pipelines

Note, “Yes” indicates the platform provides an integrated, end-to-end module (analysis + reporting) for the feature; “Limited” indicates partial/species-dependent support or external-tool reliance; and “No” indicates the feature is not provided natively. System prerequisites and installation instructions (Java, Cromwell, Docker) are provided in the project README on GitHub.

Both rMAP-2.0 and Bactopia support modular, end-to-end analysis of Illumina paired-end bacterial WGS data, including AMR, MLST/cgMLST, phylogeny, and pangenome analysis. They differ mainly in how results are packaged and consumed. rMAP-2.0 is implemented as modular WDL tasks executed via Cromwell and is designed to produce a single consolidated, navigable HTML report that integrates QC, assembly, typing, AMR, and downstream summaries into one interface (example report: https://gmboowa.github.io/rMAP-2.0/eskapee/klebsiella/report.html). In contrast, Bactopia is implemented as a modular Nextflow DSL2 pipeline that emphasizes structured per-sample outputs plus merged summary tables (e.g. AMRFinderPlus, MLST), with QC reports (e.g. FastQC) and optional Nextflow run reports for resource accounting useful for bioinformatics teams who prefer file-based outputs and downstream customization.

To provide an objective benchmark under consistent compute conditions, we measured end-to-end wall time on the same machine for a small dataset of five Illumina paired-end *E. coli* samples (test_data; NCBI/SRA; typical *E. coli* genome ≈5.0 Mb, with expected strain-to-strain variation). All runs were executed on a MacBook Pro laptop (2.3 GHz 8-core Intel Core i9, 16 GB DDR4 2667 MHz) running macOS Sequoia 15.7.1. Under these conditions, rMAP-2.0 completed the analysis and generated its consolidated HTML report in ∼3 h 12 m, while Bactopia completed the same five-sample analysis in 3 h 25 m wall time (Nextflow “Duration”).

To provide scaling context beyond the five-isolate comparison, we also recorded rMAP-2.0 wall-clock runtimes across larger dataset tiers: medium (11 *P. aeruginosa* genomes; typical genome ≈6.3 Mb; ∼3 h 50 m) and large (20 *K. pneumoniae* genomes; typical genome ≈5.5 Mb; ∼4 h 50 m). Together, these wall-time measurements provide a concise comparison of workflow ecosystems, reporting outputs, and expected runtimes for typical bacterial WGS use cases across common pathogen and cohort sizes.

### 2.3 Building a local ESKAPEE BLAST database from RefSeq

To support fully offline execution and data sovereignty, rMAP-2.0 can optionally use locally built BLAST databases for ESKAPEE pathogens. Users may either download a pre-built database archive or construct a custom database from RefSeq genomes; full instructions and example commands are provided in the GitHub documentation (https://github.com/gmboowa/rMAP-2.0).

### 2.4 Minimum sample requirements

Pangenome inference requires ≥2 samples and phylogenetic inference require ≥4 samples; below these thresholds the corresponding modules are not executed.

### 2.5 Workflow input configuration (JSON file)

Execution of rMAP-2.0 requires a JSON configuration file specifying input reads and module control flags. At minimum, paired-end Illumina FASTQ files must be provided. A simplified illustrative example is shown to demonstrate the structure of the configuration; complete, executable JSON examples are provided in the GitHub repository.

{

"rMAP.input_reads": ["sample_R1.fastq.gz", "sample_R2.fastq.gz"],

"rMAP.do_assembly": true,

"rMAP.do_variant_calling": false

}

Complete runnable examples are documented in the repository (https://github.com/gmboowa/rMAP-2.0).

### 2.6 Annotation and antimicrobial resistance genes (ARG) detection

After read QC, trimming, and genome assembly, rMAP 2.0 performs genome annotation using Prokka (Seemann 2014) generating standard annotation outputs (e.g. GFF/GenBank/FAA/FFN) that support downstream interpretation and reporting. For antimicrobial resistance gene (ARG) detection, rMAP 2.0 screens the assembled contigs using ABRicate against the ResFinder database (Florensa *et al.* 2022), which provides curated resistance gene sequences for acquired AMR determinants. In our implementation, ResFinder is used as the default ARG database (e.g. supplied as a locally installed database via the input JSON such as rMAP.local_amr_db in the example configuration).

ABRicate reports candidate ARG hits based on minimum alignment percent identity and percent coverage thresholds (ABRicate—minid and—mincov). rMAP 2.0 applies explicit identity/coverage cutoffs (defaults recorded in the workflow configuration and report), and users can adjust these thresholds either (i) by overriding the corresponding parameters via the input JSON when exposed (recommended for routine runs) or (ii) by editing the ABRicate command flags in the WDL for deeper customization, with changes tracked via version control to preserve reproducibility.

The core phylogeny analysis in our workflow was implemented through the CORE_PHYLOGENY task, which constructs robust phylogenetic trees from multiple sequence alignments. This module is designed with built-in safeguards to ensure reproducibility and data integrity. Prior to tree reconstruction, the workflow validates the alignment input, checking for file existence, content completeness, and a minimum sequence threshold (≥4 sequences). Automated logging captures runtime parameters and system information, supporting traceability and debugging. Phylogenetic inference is performed using FastTree (v2.1.11) (Price *et al.* 2010) under a generalized time-reversible (GTR) nucleotide substitution model with gamma-distributed rate heterogeneity. Bootstrap resampling (default 100 replicates) provides statistical support for tree topology, with adaptive fallback strategies (reduced replicates or minimal tree generation) implemented to handle out-of-memory or runtime errors. The final output includes a Newick-formatted phylogenetic tree, detailed log files, and execution metadata, enabling integration into downstream comparative genomics and evolutionary analyses.

The phylogenetic trees generated by the workflow are rendered using the TREE_VISUALIZATION module, which provides high-resolution, graphical outputs from Newick-formatted trees. This task employs the ETE3 (Huerta-Cepas *et al.* 2016) library with customizable parameters for tree layout (rectangular, circular, or fan), image dimensions, branch thickness, color schemes, tip labeling, and background settings. To ensure robustness across computing environments, the module supports offscreen rendering and includes fallback mechanisms to Biopython’s Phylo and matplotlib in cases where ETE3 rendering fails. Final outputs include high-quality tree images (PNG format by default), enabling seamless integration of phylogenetic results into downstream comparative genomics and publication workflows.

## 3. Results

### 3.1 Workflow runtime performance

rMAP-2.0 produces a broad range of outputs including trimming statistics, genome assembly metrics, functional annotation, variant discovery, antimicrobial-resistance and virulence profiling, mobile-genetic-element detection, multilocus sequence typing, pangenome analysis, and phylogenetic reconstruction complemented by integrated visualization and reporting. In a representative run, end-to-end analysis of 20 clinical bacterial isolates (average genome size ∼5.5 Mb) completed in ∼4.5 hours on hardware with the specifications of an average laptop computer (2.3 GHz 8-core Intel Core i9, 16 GB DDR4 RAM).

### 3.2 AMR surveillance outputs

To demonstrate rMAP 2.0’s utility for routine AMR surveillance, we report outputs that directly support surveillance interpretation at both the antimicrobial resistance determinant and lineage levels. Across the evaluated isolates, rMAP 2.0 generated standardized AMR profiling reports by screening assembled contigs against the ResFinder ARG reference database and summarizing detected genes per sample alongside alignment confidence metrics (percent identity and percent coverage) using user-defined thresholds (e.g. 90% identity and 80% coverage in the representative run) (https://gmboowa.github.io/rMAP-2.0/). These per-isolate ARG callsets provide a directly comparable, auditable basis for tracking the presence/absence of priority resistance determinants over time and across sites, and for aggregating resistance markers by organism, facility, or sampling period.

In parallel, rMAP 2.0 produced lineage-defining outputs that contextualize ARG findings within transmission- and outbreak-relevant population structure. Specifically, the workflow reported species-specific MLST sequence types (STs) and allele profiles for each isolate, enabling surveillance teams to monitor the emergence and spread of high-risk clones and to stratify ARG patterns by lineage. Where enabled, phylogenetic outputs further supported lineage investigation by providing isolate relatedness summaries that can be used to flag clusters requiring follow-up. Together, the combined ARG profiles (ResFinder) and lineage assignments (MLST/phylogeny) deliver the core outputs required for actionable AMR surveillance: detection of resistance determinants, attribution to circulating lineages, and consistent reporting suitable for longitudinal monitoring and cross-laboratory comparison.

### 3.3 End-to-end integrated reporting and visualization

**Figure 2:**
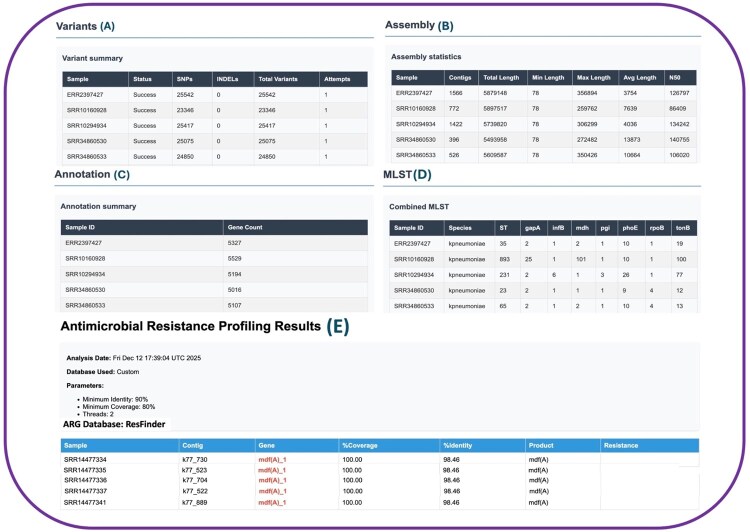
This shows the consolidated rMAP-2.0 results generated from the small test_data benchmark set of five *Escherichia coli* Illumina paired-end WGS datasets (NCBI/SRA). (A) Variant-calling summary showing per-sample SNP, INDEL, and total variant counts; all 20 clinical isolates were processed successfully. (B) Assembly statistics (contig number, total length, N50) illustrating variability in assembly quality across samples. (C) Annotation summary reporting predicted gene counts per isolate. (D) Species-specific MLST results with ST assignments and allele profiles. MLST. *In silico* multilocus sequence typing was performed from the draft assemblies using the mlst tool (Torsten Seemann), which scans contigs against PubMLST curated schemes to report the sequence type (ST) and the allelic profile for each locus. By default, the workflow auto-selects the best-matching scheme; advanced users may override the scheme (e.g. by specifying a PubMLST scheme name in the inputs/JSON). MLST may return partial profiles or no ST when assemblies are highly fragmented, loci are missing, or the appropriate scheme is unavailable. (E) Antimicrobial resistance gene (ARG) detection summary produced by screening assembled contigs against the ResFinder ARG reference database. Hits are reported per isolate with gene name and corresponding alignment metrics (% coverage and % identity); in this run, ARG calls were filtered using minimum thresholds of 90% identity and 80% coverage (parameters shown in the panel), ensuring that only high-confidence ResFinder matches are retained for reporting.

**Figure 3:**
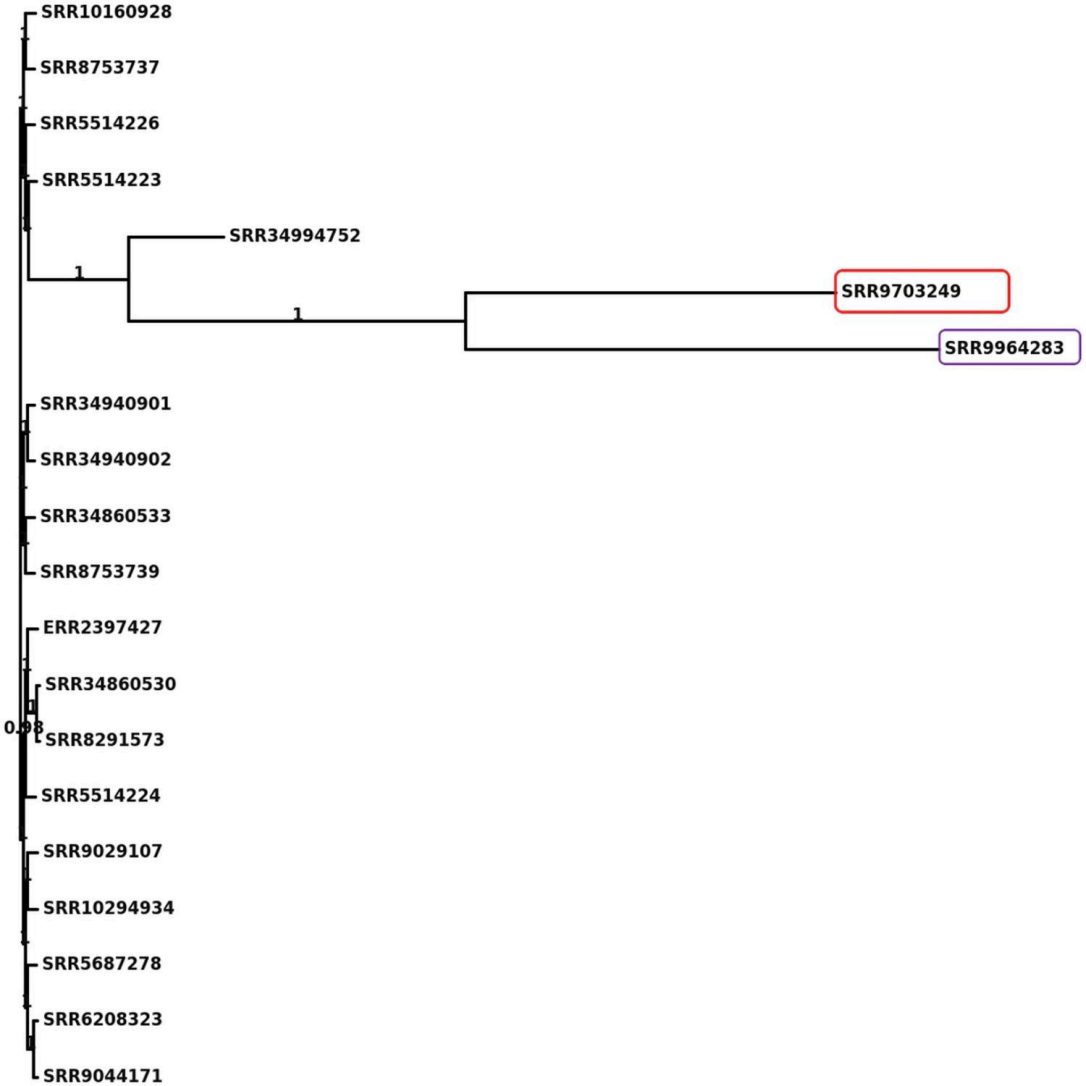
Core-gene phylogeny of the large benchmark dataset (*n *= 20 genomes; primarily *Klebsiella pneumoniae*). Maximum-likelihood tree inferred from the concatenated core-gene alignment of 20 clinical Enterobacterales isolates using a GTR + Γ model with 100 bootstrap replicates (support values shown at key nodes). Most samples cluster as *K. pneumoniae*, forming several tightly related clades. Two isolates SRR9703249 (*E. cloacae*, ST182) and SRR9964283 (*Escherichia coli*, ST131) form a distinct, well-supported branch, consistent with their MLST species assignments and highlighting the presence of mixed-species samples within the large dataset. Overall, the topology summarizes relatedness among *K. pneumoniae* isolates while clearly separating the non*-Klebsiella* outgroups.

**Figure 4:**
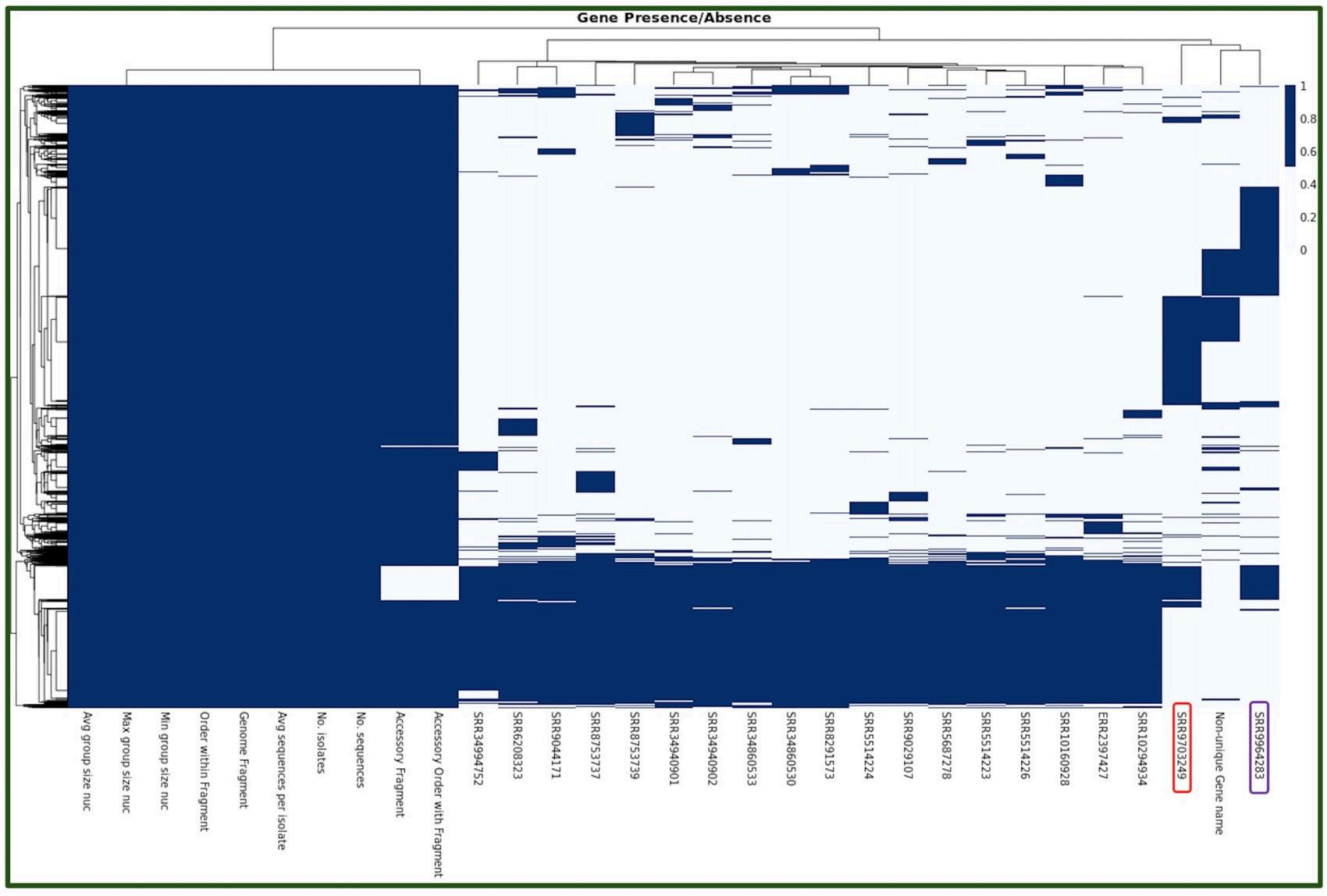
Gene presence–absence across clinical isolates. Heatmap of the pangenome showing binary gene presence and absence across 20 genomes (columns), with genes clustered by similarity (rows) and isolates hierarchically clustered by gene-content profiles (top dendrogram). Klebsiella pneumoniae isolates group into closely related clusters with a large, shared core and structured accessory variation. In contrast, SRR9703249 (originally labeled in NCBI/SRA as K. pneumoniae but identified here by MLST as Enterobacter cloacae ST182) and SRR9964283 (Escherichia coli ST131) segregate at the extreme right with extensive gene-content differences, consistent with the core-gene phylogeny. These two non-Klebsiella isolates are highlighted in the figure for clarity. This reclassification of SRR9703249 corrects its original SRA metadata and highlights conserved genomic backgrounds among K. pneumoniae versus distinct accessory repertoires in the non-Klebsiella outliers.

rMAP-2.0 generates a comprehensive suite of outputs (Figs 2–4; Table 4) trimmed reads; assembled genomes (FASTA); variant calls (VCF); annotations (GFF/GenBank); gene/sequence files (FFN/FSA); alignments (BAM/BED); summary tables (TSV/CSV); heatmaps and phylogenetic trees (Newick with PNGs); plus, task-specific HTML reports. For each batch of isolates/samples run, the tool compiles all outputs into a single interactive HTML report that aggregates results from every analysis step and provides a user-friendly, shareable interface. An example is available at (https://gmboowa.github.io/rMAP-2.0/), with per-sample trimming/QC, assembly statistics, variant summaries, annotation, pangenome results, MLST, AMR/MGE/virulence/BLAST outputs, and phylogenetic trees, all navigable via a structured sidebar and linked subsections.

**Table 4:** Outputs by modules in rMAP-2.0.

Module	Key output files
**TRIMMING**	Trimmed FASTQ files (*.*fastq.gz*)
**QUALITY_CONTROL**	MultiQC reports; FastQC outputs (*.zip, *.html)
**ASSEMBLY**	Assembled contigs (*.*fasta*)
**VARIANT_CALLING**	Variant call format files (*.*vcf*)
**AMR_PROFILING**	Resistance profiles (*.*txt*, *.*tsv*)
**MLST**	MLST profiles (*.*txt*, *.*tsv*)
**MGE_ANALYSIS**	Mobile genetic element annotations (*.*txt*, *.*tsv*)
**VIRULENCE_ANALYSIS**	Virulence gene predictions (*.*txt*, *.*tsv*)
**ANNOTATION**	Genomic feature annotations (*.*gff*, *.*gbk*)
**BLAST_ANALYSIS**	Top BLAST hits (*.*tsv*, *.*xml*)
**PANGENOME**	Roary outputs: gene_presence_absence.*csv*; core_gene_alignment.*aln*
**ACCESSORY_PHYLOGENY**	Phylogenetic tree for accessory genes (*.*nwk*)
**CORE_PHYLOGENY**	Core genome tree and alignment files (*.*nwk*)
**TREE_VISUALIZATION**	Rendered tree visualization (*.*png*)
**MERGE_REPORTS**	Consolidated report (final_report.*html*), assets (assets/*), combined summaries (*.*tsv*, *.*csv*), pipeline summary (pipeline_summary.*json*)

For deeper exploration, all intermediate and detailed outputs covering trimming, quality control, assembly, annotation, variant calling, BLAST, antimicrobial resistance profiling, mobile genetic element detection, MLST, virulence profiling, pangenome analysis, and phylogenetic reconstruction and visualization are retained in their respective task call directories. By structuring results into standardized, linkable sections (Trim, QC, Assembly, Annotation, Pangenome, MLST, Variants, AMR, MGE, Virulence, BLAST, Phylogeny) with consistently labeled per-sample pages and structured readable tables, the consolidated HTML report enhances interoperability with LIMS and dashboards while delivering interpretable, actionable summaries for public-health and clinical end-users.

### 3.4 Antimicrobial resistance gene profiling

Using the rMAP-2.0 AMR module, we screened assembled contigs from a representative *K. pneumoniae* isolate (ERR2397427) drawn from the large benchmark tier (*n *= 20, *K. pneumoniae* genomes) against the resistance database, applying minimum identity and coverage thresholds of 90% and 80%, respectively (https://gmboowa.github.io/rMAP-2.0/eskapee/klebsiella/assets/sections/ERR2397427_amr.html). The analysis identified multiple acquired resistance determinants spanning several antimicrobial classes, including the aminoglycoside-modifying enzyme *aadA2* (streptomycin resistance); the extended-spectrum β-lactamases *blaCTX-M-55* and *blaSHV-33*, consistent with reduced susceptibility to β-lactams and third-/fourth-generation cephalosporins (e.g. amoxicillin, ampicillin, aztreonam, cefepime, cefotaxime, ceftazidime, ceftriaxone, piperacillin, ticarcillin); the phenicol resistance gene *floR*; the fosfomycin resistance gene *fosA6*; and the lincosamide resistance determinant lnu(F). Fluoroquinolone resistance was supported by the efflux pump components *oqxA*/*oqxB* and the plasmid-mediated quinolone resistance gene *qnrS1*, while tetracycline resistance was mediated by tet(A). Together, this profile is consistent with a multidrug-resistant genotype in ERR2397427 and illustrates how rMAP-2.0 reports per-gene alignment metrics (percent identity and percent coverage) to support transparent, auditable interpretation of resistance calls.

In the large benchmark dataset (*n *= 20 genomes; report: https://gmboowa.github.io/rMAP-2.0/eskapee/klebsiella/report.html), the MLST table indicates that the two divergent tips in the core-gene phylogeny (Fig. 3) correspond to SRR9703249 and SRR9964283, typed as *Enterobacter cloacae* (ST182) and *E. coli* (ST131), respectively. The remaining samples are predominantly *K. pneumoniae* with diverse sequence types (STs). These non-*Klebsiella* assignments explain the long, separate branch in the core-gene tree, with SRR9703249 and SRR9964283 effectively serving as outgroups to the main *K. pneumoniae* cluster. Notably, SRR9703249 was deposited in NCBI/SRA as *K. pneumoniae* but is consistently identified by MLST as *E. cloacae* (ST182) and clusters separately in the phylogeny. This discrepancy underscores the value of genome-based workflows for accurate species identification and highlights how metadata errors in public repositories can propagate into downstream surveillance and epidemiological analyses.

Integrating phylogenetic reconstruction with gene presence/absence heatmaps provides a powerful framework for dissecting both evolutionary relationships and functional diversity among bacterial clinical isolates. While the core-gene phylogeny (Fig. 3) establishes the evolutionary relatedness of isolates, the pangenome heatmap (Fig. 4) complements this by revealing the distribution of accessory and strain-specific genes that underlie phenotypic variability. Together, these approaches distinguish clonal clusters of *K. pneumoniae* with highly conserved gene content from divergent species, such as SRR9703249 (*E. cloacae*, ST182) and SRR9964283 (*E. coli*, ST131), which segregate both phylogenetically and at the level of gene repertoire. This combined perspective not only validates species-level assignments but also highlights the functional implications of genomic divergence, thereby strengthening epidemiological interpretation and guiding downstream analyses and interpretation of antimicrobial resistance and virulence determinants.

### 3.5 Performance across ESKAPEE taxa

To assess performance across the ESKAPEE group, we ran rMAP-2.0 on multiple isolates representing *K. pneumoniae*, *A. baumannii*, *Enterobacter spp*., *E. faecium*, *E. coli*, *P. aeruginosa*, and *S. aureus*, and we provide the resulting interactive HTML reports online (https://gmboowa.github.io/rMAP-2.0/). Across these taxa, rMAP-2.0 consistently generated the same core outputs in a standardized report format, including QC/trimming, assembly metrics, annotation, MLST, variant calling, AMR/MGE/virulence profiling, BLAST, and phylogenetic inference. rMAP-2.0 is optimized and validated for isolate whole-genome sequencing (pure cultures) rather than complex metagenomic samples; comprehensive metagenomic support (multi-organism profiling and abundance-aware reporting) is planned for future work.

### 3.6 HPC and cloud execution

rMAP-2.0 is implemented in WDL and executed with Cromwell, enabling portable deployment across local workstations, HPC schedulers, and cloud backends while preserving consistent task structure and outputs. In addition to the laptop benchmarks reported above, we validated end-to-end execution in both an HPC environment and a cloud backend, confirming reproducible runs with the same organization output and consolidated reporting across compute settings. Wall-clock runtime varies with read depth, database version/size, and available compute and storage throughput; however, Cromwell supports straightforward scaling via per-isolate parallelization and modular task execution without requiring workflow rewrites.

## 4 Discussion

rMAP 2.0 addresses a critical gap in microbial genomics by reducing dependency and reproducibility challenges that hinder many existing workflows. Its streamlined modular design empowers public health microbiology laboratories in low- and middle-income countries to conduct advanced, reproducible analyses of ESKAPEE pathogens. By adopting a WDL–Cromwell–Docker architecture, the pipeline enables seamless execution across heterogeneous environments, from personal workstations to high-performance computing clusters and cloud platforms. This design allows laboratories in both high-resource and resource-limited settings to perform reproducible, end-to-end genomic analyses of ESKAPEE pathogens without the burden of installing and maintaining complex software stacks.

Compared with several bacterial genomics platforms such as Pathogenwatch (https://pathogen.watch/), AMR Watch (https://amr.watch/), Microreact (https://microreact.org/), PATRIC (https://www.bv-brc.org/view/Bacteria/), AMRnet (https://www.amrnet.org/), and EnteroBase (https://enterobase.warwick.ac.uk/), rMAP 2.0 stands out for its modular design, transparent containerization, scalability, and flexibility across both cloud-native and fully offline settings. Its ability to run entirely within institutional or local environments directly addresses data privacy and sovereignty concerns that limit adoption of web-based tools, making it especially valuable for public health laboratories handling sensitive genomic and epidemiological datasets.

rMAP 2.0 generates output covering QC and trimming reports, genome assemblies, and variant calls, as well as antimicrobial resistance, plasmid, and virulence profiling, MLST typing, pangenome reconstruction, and phylogenetic trees. Together, these results provide a comprehensive foundation for microbial genomic surveillance and support diverse applications, including outbreak investigations, resistance-gene tracking, and integration with platforms such as the Interactive Tree of Life (iTOL) (https://itol.embl.de/). By providing standardized, containerized workflows, rMAP 2.0 fosters reproducibility and facilitates data comparability across laboratories and regions an important requirement for transnational AMR surveillance efforts.

Despite these strengths, some limitations remain. The workflow is currently optimized for Illumina short-read data. Moreover, while Docker ensures strong reproducibility, some HPC environments restrict container use, which may require alternative container solutions (e.g. Singularity/Apptainer) in future versions of rMAP 2.0. Although rMAP 2.0 streamlines installation and execution via containers and WDL, routine use still requires basic command-line proficiency and JSON editing; future work will explore GUI-based wrappers to further lower the barrier for non-specialist users. Finally, while the pipeline leverages widely used reference databases for AMR, virulence, and mobile genetic elements, keeping these resources up to date remains an ongoing challenge for genomic surveillance tools (Sherry *et al.* 2025). Recognizing these limitations provides a pathway for future enhancement.

## 5 Conclusion

rMAP 2.0 provides a practical, reproducible framework for microbial whole-genome analysis of ESKAPEE pathogens, combining workflow standardization with containerization to improve portability across local, HPC, and cloud environments. In this study, we demonstrate its ability to generate core outputs relevant to AMR monitoring such as ARG profiles and lineage assignments in a consistent and auditable manner. However, we did not conduct a formal AMR surveillance study; rather, rMAP 2.0 is intended to support surveillance when integrated into ongoing laboratory and public health programs.

Looking ahead, sustainability and scalability will depend on continued versioned releases, database refresh strategies, and interoperability with established analysis ecosystems (e.g. Terra/WDL/Cromwell) to enable collaborative, population-scale analyses while maintaining data-governance requirements. Future work will expand support for long-read and hybrid sequencing, strengthen plasmid and mobile-element resolution, and explore user-friendly interfaces to reduce remaining barriers (e.g. command-line use and JSON configuration) for non-specialist users.

Overall, rMAP 2.0 is positioned as enabling infrastructure for standardized microbial genomics that can be adopted within national and regional AMR programs to accelerate the translation of sequencing data into actionable public health insight.

## Data Availability

The data underlying this article are available in the ENA/NCBI SRA under the following BioProjects: PRJNA1179472 (SRR34940901, SRR34940902); PRJNA1301420 (SRR34860530, SRR34860533); PRJNA1306097 (SRR34994752); PRJNA526408 (SRR9044171); PRJNA544438 (SRR10294934); PRJNA555206 (SRR9703249); PRJNA526349 (SRR9964283); PRJNA557813 (SRR10160928); PRJEB19226 (ERR2397427); PRJNA508495 (SRR8753737, SRR8753739, SRR8291573); PRJNA523709 (SRR9029107); PRJNA351846 (SRR5514223, SRR5514224, SRR5514226, SRR5687278); PRJNA413040 (SRR6208323); PRJNA727368 (SRR14477334, SRR14477335, SRR14477336, SRR14477337, SRR14477341).
